# One-step purification of two novel thermotolerant β-1,4-glucosidases from a newly isolated strain of *Fusarium chlamydosporum* HML278 and their characterization

**DOI:** 10.1186/s13568-020-01116-1

**Published:** 2020-10-08

**Authors:** Yongling Qin, Qiqian Li, Fengfeng Luo, Yue Fu, Haiyan He

**Affiliations:** 1 College of Chemistry and Biological Engineering, Hechi University, 546300 Yizhou, China; 2 Guangxi Colleges Universities Key Laboratory of Exploitation and Utilization of Microbial and Botanical Resources, 546300 Yizhou, China

**Keywords:** *Fusarium chlamydosporum* HML278, β-glucosidase, purification, heat-resistant, transglycosidic activity

## Abstract

A newly identified cellulase-producing *Fusarium chlamydosporum* HML278 was cultivated under solid-state fermentation of sugarcane bagasse, and two new β-glucosides enzymes (BG FH1, BG FH2) were recovered from fermentation solution by modified non-denaturing active gel electrophoresis and gel filtration chromatography. SDS-PAGE analysis showed that the molecular weight of BG FH1 and BG FH2 was 93 kDa and 52 kDa, respectively, and the enzyme activity was 5.6 U/mg and 11.5 U/mg, respectively. The optimal reaction temperature of the enzymes was 60 ℃, and the enzymes were stable with a temperature lower than 70 ℃. The optimal pH of the purified enzymes was 6.0, and the enzymes were stable between pH 4–10. *K*_m_ and *V*_max_ values ​​were 2.76 mg/mL and 20.6 U/mg for pNPG, respectively. Thin-layer chromatography and high-performance liquid chromatography analysis showed that BG FH1and BG FH2 had hydrolysis activity toward cellobiose and could hydrolyze cellobiose into glucose. In addition, both enzymes exhibited transglycoside activity, which could use glucose to synthesize cellobiose and cellotriose, and preferentially synthesize alcohol. In conclusion, our study demonstrated that *F. chlamydosporum* HML278 produces heat-resistant β-glucosidases with both hydrolytic activity and transglycosidic activity, and these β-glucosidases have potential application in bioethanol and papermaking industries.

## Key points

- Two new β-glucosides enzymes of Fusarium chlamydosporum HML278 were purified.
- The enzymes were stable under 70 ℃ and exhibited transglycoside activity.
- The enzymes have potential application in bioethanol and papermaking industries.

## Introduction

Lignocellulose is a linear polysaccharide linked by D-glucose via β-(1,4)-glycosidic bonds, and is the most abundant renewable resource on earth (Zhang et al. 2010; Kovacs et al. 2009; Sánchez and Cardona 2008). Lignocellulose can eventually be degraded into glucose under the synergy of the cellulase system: endoglucanase (EC 3.2.1.4) randomly acts on the non-crystalline region inside the cellulose molecule to produce glucose and short fiber oligosaccharides sugar; Exoglucanase (EC 3.2.1.91) hydrolyzes β-1,4-glycosidic bonds from outside to inside along the non-reducing end of cellulose to release cellooligosaccharides, cellobiose or glucose; β-glucosidase (EC 3.2 0.1.21) hydrolyzes cellobiose or other soluble cellobiose and cellooligosaccharides into glucose (Gomes et al. 2018; Fan et al. 2016; Arantes and Saddler 2010 ).

β-glucosidase plays an important role in the hydrolysis of cellulose. The intermediate products of cellulose hydrolysis, such as cellobiose, cellooligosaccharides, have a strong inhibitory activity on the activity of exoglucanase and endoglucanase. β-glucosidase hydrolyzes cellobiose and cellooligosaccharides to produce glucose, reducing the inhibitory effect of these intermediate products on exoglucanase and endoglucanase, thus improve the saccharification rate of cellulose enzymes (Gomes et al. 2018; Kamila et al. 2016; Chauve et al. 2010; Ikeda et al. 2006; Tanaka et al. 2006; Wen et al. 2005).

β-glucosidase is an important industrial enzyme that has been used in many bioprocesses, including the processing of biofuels, paper industry, textile industry, waste, and food (Pei et al. 2012; Tian et al. 2010; Bayer et al. 2008; Han and Chen 2008; Rubin 2008; Villena et al. 2007; Zaldivar et al. 2001).

Thermophilic fungi can produce a variety of hydrolytic enzymes that hydrolyze cellulosic substances. These enzymes are produced in high yield and exhibit good catalytic performance stability, and are a promising industrial enzyme. (Zhang et al. 2014; Haven and Jørgensen 2013; Prawitwong et al. 2013; Pei et al. 2012).

Screening of strains producing ezymes with high β-glucosidase activity is very important in industry for the comprehensive utilization of cellulose resources and other application (Miettinen-Oinonen et al. 2004; Saloheimo et al. 1997). Xylanase, endoglucanase, and other cellulose hydrolyzing enzymes produced from *Fusarium* sp. have high cellulose degradation activity, and these enzymes exhibited synergistic effect on of cellulose into ethanol (Gómez-Gómez et al. 2001; Kumar et al. 1991).

In our previous study, a heat-resistant cellulase-producing *Fusarium chlamydosporum* strain HML278 was screened from the virgin forest samples in Guangxi, China. This strain was shown to produce and secrete three major enzyme components of cellulase system, including endoglucanase, microcrystalline cellulose, and β-glucosidase and xylanase (Qin et al. 2010). In this study, two new β-glucosidases with hydrolytic and transglycosidic activities from *F. chlamydosporum* HML278 were rapidly isolated and purified by using improved non-denaturing active gel electrophoresis combined with gel filtration chromatography. The optimal reaction temperature of the enzymes was 60 ℃, and the enzymes were stable below 70 ℃. The new identified enzymes may have great potential applications in bioethanol and papermaking industries.

## Materials and methods

### Strain

The cellulase-producing *F. chlamydosporum* HML 278 strainwas used in this study. *F. chlamydosporum* HML 278 was originally isolated from the soil beneath the rotten wood in Mulun Forestry Center, Huanjiang County, Guangxi, China (Qin et al. 2010) and deposited in the Chinese Center for Type Culture Collection (Accession No. CCTCC AF 2020006).

### Production of cellulase by solid-state fermentation and enzymatic activity test

*Fusarium chlamydosporum* HML278 was maintained on PDA medium at 4 ℃ in Guangxi Colleges Universities Key Laboratary of Exploitation and Utilization of Microbial and Botanical Resources.

Production of cellulase by solid-state fermentation: the screened cellulase-producing strain grown on PDA slant was washed off with 10 mL of physiological saline to make a spore solution, and 10^7^ spores were transferred to the solid medium for second round of screening for cellulase. To make the solid medium, 6 g bagasse, 4 g bran, and 30 ml Mandels nutrient solution (Kwon et al. 1994) were mixed in 500 ml erlenmeyer flask. The flask was flipped twice a day, and the strain was grown for 4 days at 30 °C. 200 mL of sterile ddH_2_O was added to the culture, and was further leached at 40 °C in a constant temperature water bath for 1 hour. The culture was filtered with four layers of gauze, and centrifuged at 6000 r/min for 10 min. The supernatant containing crude enzymes was collected and stored at 4 °C until use (Qin et al. 2010).

Detection of β-Glucosidase enzyme activity: 0.02 M citric acid-sodium citrate buffer solution (pH 4.8) was used to prepare 1% salicin (Fluka Chemical Corp, USA) solution substrate. 0.05 mL of enzyme solution with appropriate concentration was mixed with 1 mL of 1% salicin solution, and the reaction was carried out at 60 °C for 30 min. 3 mL of DNS reagent was added to stop the reaction. The reaction solution was boiled for 6 min, followed by incubating at cold water bath. The absorbance was measured at 540 nm. The amount of enzyme that produces 1 µmol of glucose per minute was defined as 1 unit of enzyme activity (U) (Shoemaker and Brown 1978).

## Rapid detection of β-glucosidase enzyme activity

The plate used for rapid detection of β-glucosidase enzyme activity (Kwon et al. 1994) was made with following components: ferric chloride 0.03%, aescin 0.1%, agar 1.5%.

### Detection of soluble total protein

The protein concentration was measured at 595 nm withthe Bradford method (Bradford 1976) by using a Bradford Protein Assay Kit (Beyotime Institute of Biotechnology, China).

### Purification of β-glucosidase

All purification steps were performed at 4 °C.

Active recovery of non-denaturing gel electrophoresis: The non-denaturing gel consisting of 8% separation gel and 4% stacking gel was run at 50 V constant voltage at 4 °C. After electrophoresis, the activity of β-glucosidase in the gel was detected by staining of gel with specific substrate (Kwon et al. 1994) containing 0.03% FeCl_3_ and 0.1% aescin. After staining for 1 min at 30 °C, the gel was immediately rinsed with distilled water to stop the reaction. The active protein band with black precipitation was cut off, and grinded in a pre-cooled mortar. The sample was leached with citric acid-citrate buffer (20 mM, pH 4.8) at 4 °C for 12 h, and centrifuged at 4000 r/min for 20 min in a 5000 Da ultrafiltration tube for concentration and desalting.

The enzyme was further purified by HiPrep 16/60 Sephacryl S-200 h\High Resolution gel filtration chromatography column, using a BioLogic DuoFlow Pathfinder 80 purifier system (pressure 73 psi). The enzyme was eluted by using elution buffer containing 0.05 mol/L PBS and 0.15 mol/L NaCl (pH 7.2) at the flow rate of 1 mL/min. The enzyme activity of the purified protein was detected referring to the enzyme activity rapid detection plate of β-glucosidase, and the protein purity was detected by using SDS-PAGE.

### SDS-polyacrylamide gel electrophoresis(SDS–PAGE)

The enzyme solutions were subjected to 12% SDS-polyacrylamide gel electrophoresis, and the gel was stained with Coomassie Brilliant Blue R250. The molecular weight of purified proteins was assessed by comparing the relative mobility of purified protein with low molecular weight standard protein (Laemmli 1970).

### Zymogram analysis of purified enzyme

The collected enzyme solution from HML278 was subjected to non-denaturing protein gel electrophoresis with pH 8.3 electrophoresis buffer at 4 ℃ by using 50 V constant voltage. The separation gel and stacking gel was made by 8% acrylamide and 4% acrylamide, respectively. After the electrophoresis, the acrylamide separation gel was cut and partly stained with silver, and the other part was stained with specific substrates of different cellulases.

To analyze the activity of β-glucosidase from cut gel, the gel was active stained with staining solution containing 0.1% escin (Sigma) and 0.03% ferric chloride (Sigma) for 5 minutes at 30 ℃. The protein with β-glucosidase activity will catalyze the substrate to produce a yellow-black product (Kwon et al. 1994).

### Analysis on the hydrolysis activity of purified β-glucosidase

Experiments for analyzing HML0366 β-glucosidase enzyme hydrolysis activity and transglycoside-mediated synthesis of gentiobiose.

β-glucosidase hydrolysis assay: 10 mL of 1% (m/v) cellobiose dissolved in citrate buffer (50 mM, pH 4.8) was used as a substrate, and 2 mL of enzyme solution was added to react at 30 °C for 30 min.

High performance liquid chromatography (HPLC) analysis of sugar components: The system utilized a refractive index detector and Hanbang amino column (250 mm × 4.6 mm, 5 µm, (Hanbon Sci. & Tech. Lichospher NH2, China). 40 ℃; mobile phase: acetonitrile/Water (4: 1, v/v); flow rate: 1 mL/min; injection volume: 5 µL.

TLC method for detecting sugar components (Jo et al. 2003): Silica thin-layer chromatography detection was utilized. Expanding agent: n-butanol: ethyl acetate: ammonia: water = 6: 3: 3: 1 (v/v). Developer: A: 1 g aniline + 25 mL acetone, B: 1 mL dianiline + 25 mL acetone. After mixing A and B, 5 mL 85% phosphoric acid was added and mixed well. After the chromatography, the plate was blown dry and color developer was sprayed, and dried at 120 °C for 10 minutes to develop color.

Cellobiose was dissolved in 20 mM citrate buffer (pH 4.8), and enzyme solution was added at 100: 1 (v/v), and reacted at 30 °C for 3 h. The product was detected by thin-layer chromatography (Jo et al. 2003; Qin et al. 2011).

#### Detection and identification of proteins by tandem time-of-flight mass spectrometry

Purified enzymes were identified by tandem time-of-flight mass spectrometry: The enzyme samples were first subjected to SDS-PAGE, and the β-glucosidase band was cut out, followed by subjecting to tandem time-of-flight mass spectrometry. The fingerprints of peptide fragments were obtained after scanning analysis by time-of-flight mass spectrometry (4800 Proteomics Analyzer, Applied Biosystems, USA), and the data was analyzed by using the Mascot software to query and identify purified enzymes on the SWISS-PROT database (Scheibner et al. 2008; Lee et al. 2007).

## Enzymatic properties of purified enzyme

### The effect of temperature on the enzyme activity and stability of β-glucosidase

The definition of relative enzyme activity: the highest enzyme activity under a certain condition of the experimental project was set to 100%, and the ratio of enzyme activity under other conditions to the highest enzyme activity was defined as relative enzyme activity.

To determine the optimal temperature of endoglucanase and β-glucosidase, their enzyme activity was measured under the conditions of 30 ℃-90 ℃ in 50 mM acetate buffer of.

To determine the effect of temperature on the stability of β-glucosidase, the enzyme was incubated in a water bath at temperatures between 40 ℃ and 90 ℃ with a gradient of 5 ℃. The enzymes were incubated at each temperature for 60 min, and the residual enzyme activity was then measured at 60 ℃.

### **The impact of pH on the enzyme activity and stability of β-glucosidase**

To determine the effect of pH on enzyme activity of β-glucosidase, the following four solutions with a concentration of 50 mM were used: disodium hydrogen phosphate-citric acid buffer, pH 2.6–7.5; Tris-HCl buffer, pH7.5-pH 9.0; glycine-NaOH buffer, pH 9.0–11.0.

Under the temperature condition where the enzyme is stable, the enzyme was mixed with the buffer with a pH value ranging between 3.0 and 9.0, and the relative enzyme activities and the optimal pH value of endoglucanase and β-glucosidase were determined.

The enzyme was further stored in a solution with a pH value between 3.0 and 11.0. After being left at 4 °C for 24 h, it was kept at 30 °C for 3 hours. The relative enzyme activities of endoglucanase and β-glucosidase were determined at the optimum pH and temperature.

### Effect of metal ions on β-glucosidase activity

Different metal ions were added to the purified enzyme solution with a final concentration of 2 mM, and the enzyme activity was then tested. The enzyme activity was calculated according to the average value of data from three parallel experiments.

### Kinetics analysis of the purified β-glucosidase

To determine the kinetic parameters of the enzymatic reaction of β-glucosidase, pNPG was used as the substrate and the reaction was performed under pH 4.8 at 30 ℃. The initial reaction rate was calculated, and the *K*_m_ value and *V*_max_ of the purified β-glucosidase was calculated by using double reciprocal plotting method (Lineweaver-Burk plot (Lineweaver and Burk 1934).

## Results

### Purification and characterization of β-glucosidase from ***F. chlamydosporum*** HML278 fermented solution

**Fig. 1:**
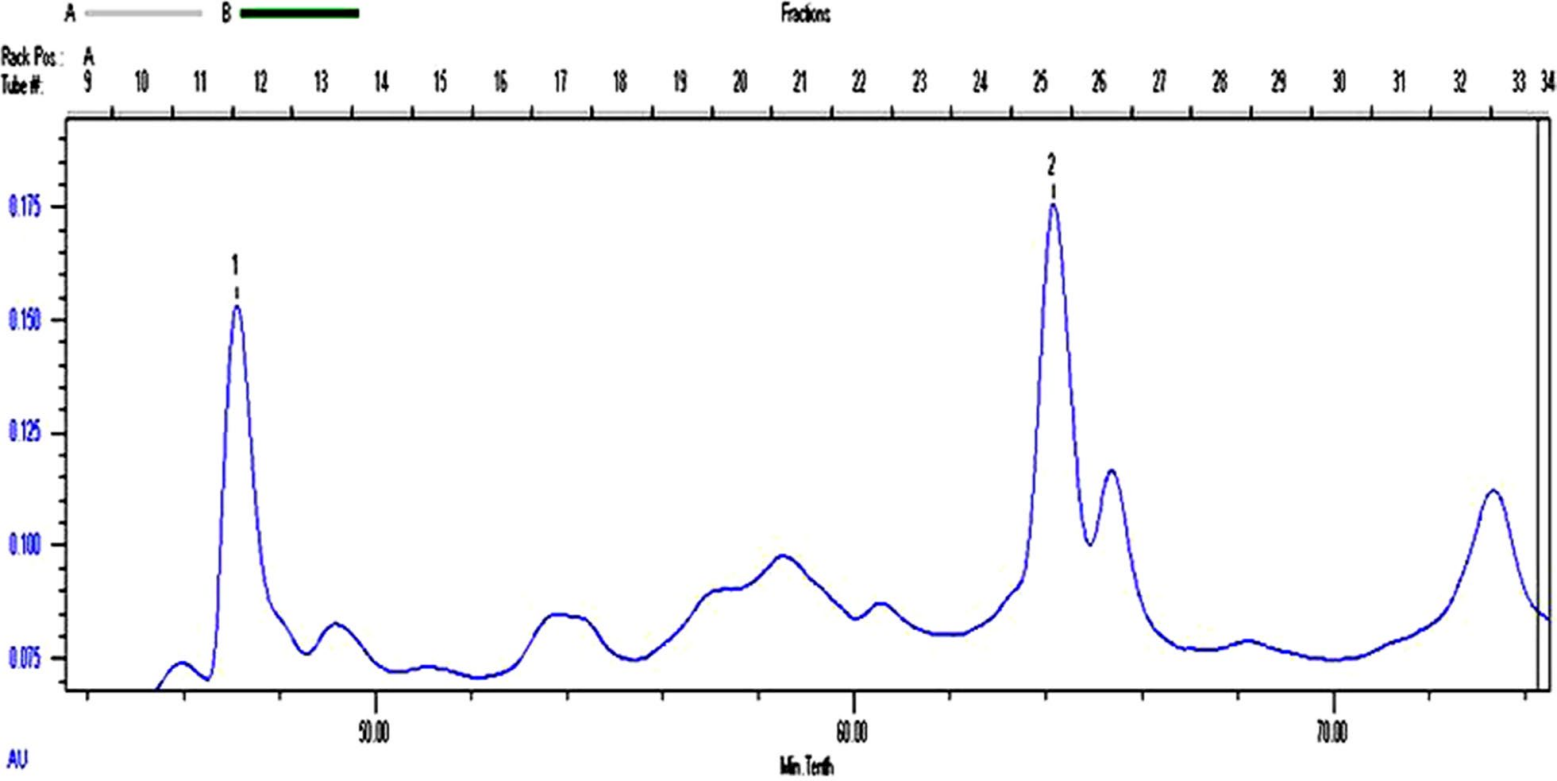
Purification of enzymes by HiPrep 16/60 Sephacryl S-200 h\High Resolution chromatography. The first protein peak was BG FH1, and the second peak was BG FH2

**Fig. 2:**
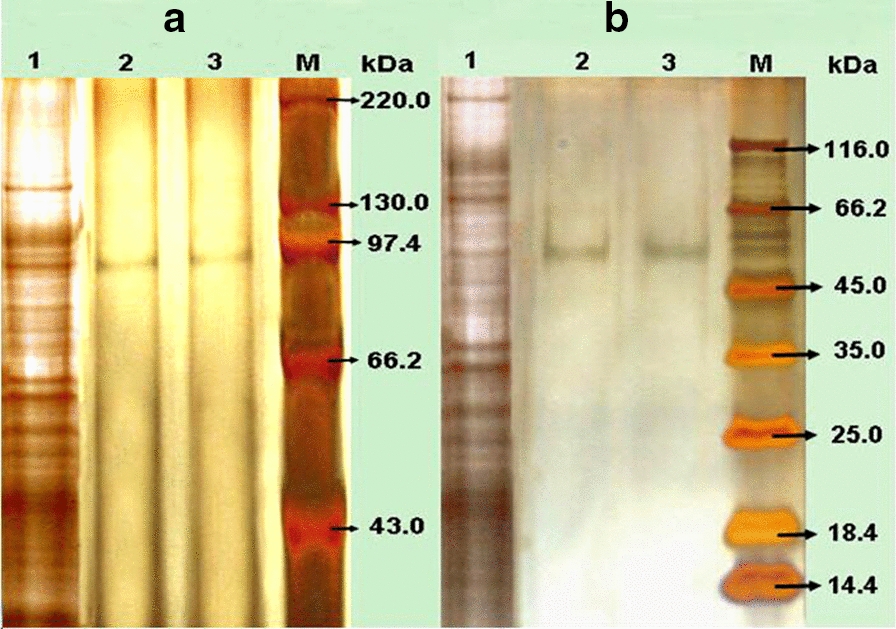
Silver staining of SDS-PAGE for purified β-glucosidases produced by *F.* *chlamydosporum* HML 278. A, BG FH1; B, BG FH2. 1 and M were original fermented solution and protein marker, respectively. 2 and 3 were purified BG FH1 and BG FH2, respectively

**Fig. 3:**
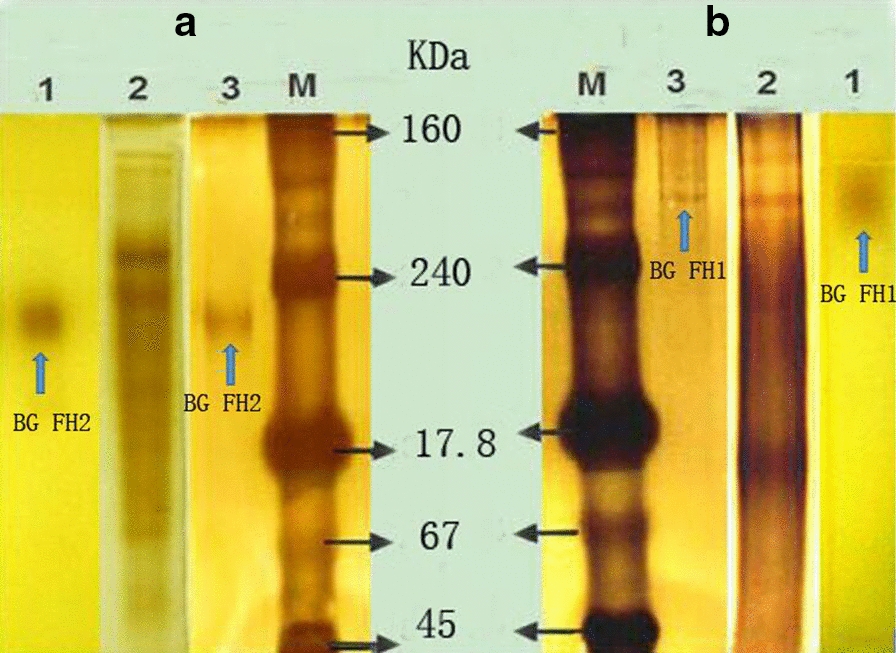
Zymogram analysis of β-glucosidases produced by *F. chlamydosporum* HML 278. A, BG FH2; B, BG FH1, respectively. Lane 1 and 3 were purified enzymes, respectively. land 2 was the original fermented solution. lane M was the Serva native-PAGE protein marker (SERVA Electrophoresis GmbH, Germany): The arrows pointed to the location of the corresponding enzymes. lane 1 was stained for β-glucosidase activity using 0.03% FeCl_3_ and 0.1% esculoside; lanes 2, 3, and M were silver stained

It was shown that the enzyme activity of β-glucosidase reached a maximum of 115.2 U/g after 4 days of solid bagasse culture of *F. chlamydosporium* HML278. To purify the enzymes from fermented solution of *F. chlamydosporium* HML278, an anion exchange column was initially utilized, but the separation effect was not promising and there was no obvious protein peak, it was speculated that the isoelectric point may be too high. A total of 115.2 U (20 mL) of crude enzyme solution was further subjected to non-denaturing gel electrophoresis without adding a comb in order to increase the sample load. The active gel was recovered, and subjected to gel filtration chromatography. The enzyme BG FH1was obtained after about 48 min, and BG FH2 was obtained about 64 min after running (Fig. 1). SDS-PAGE analysis showed that the molecular weights of BG FH1and BG FH2 were 93 kDa and 52 kDa, respectively (Fig. 2), and the recovery rate of enzymes for purification was 4.0% and 20.0%, respectively. The fold-purification of BG FH1and BG FH2 was 14.0 and 28.8 (Table 1), respectively,and the enzyme activity for each enzyme was 5.6 U/mg and 11.5 U/mg, respectively. The zymography analysis of non-denaturing electrophoresis confirmed that the strain produced two different β-glucosidases, and both enzymes are a single subunit protein (Fig. 3).

**Table 1:** Summary of purification of β-glucosidases produced by *F. chlamydosporum* HML 278

Purification step	Total activity (U)	Total protein (mg)	Specific activity (U/mg)	Purification fold Yield (%)	Purification fold
Crude enzyme	115.2	282.0	0.4	100.0	1.0
Native Page	45.6	14.8	3.1	39.6	7.8
Gel filtration chromatographic					
BG FG1	4.5	0.8	*5.6*	4.0	14.0
BG FG2	21.8	1.9	*11.5*	20.0	28.8

Activities were measured on CMC

Some other peptide sequences were detected by tandem time-of-flight mass spectrometry, but there was not any enzyme information in the protein database, thus these protein sequences were not identified.

## The hydrolysis activity of BG FH1 and BG FH2

**Fig. 4:**
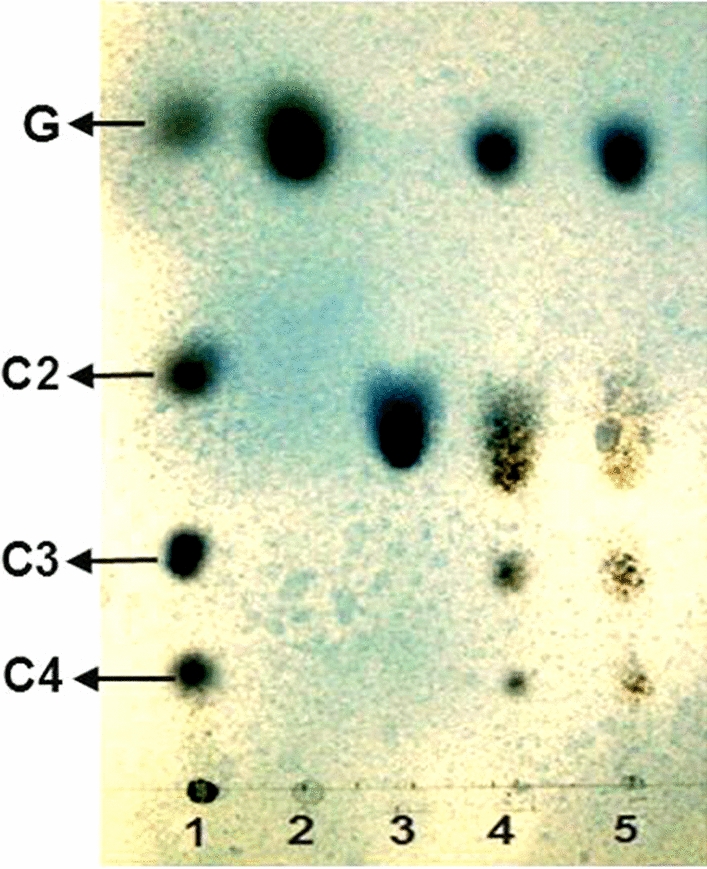
Hydrolysis property and transglycosylation activity of the purified β-glucosidases from *F. chlamydosporum* HML 278, as demonstrated by thin layer chromatography. Lane 1, mixed standards containing G Glucose, C2 Cellobiose, C3 Cellotriose, and C4 Cellotetraose; Lane 2, Glucose standard; Lane 3, Cellobiose standard; Lane 4, Cellobiose + BGFH1; Lane 5, Cellobiose + BG FH2

The thin-layer chromatography experiment showed that both BG FH1 and BG FH2 had hydrolytic activity, and can hydrolyze cellobiose to generate glucose. In addition, the enzymes also showed transglycoside activity and can synthesize cellotriose and cellotetraose using glucose (Fig. 4).

**Fig. 5:**
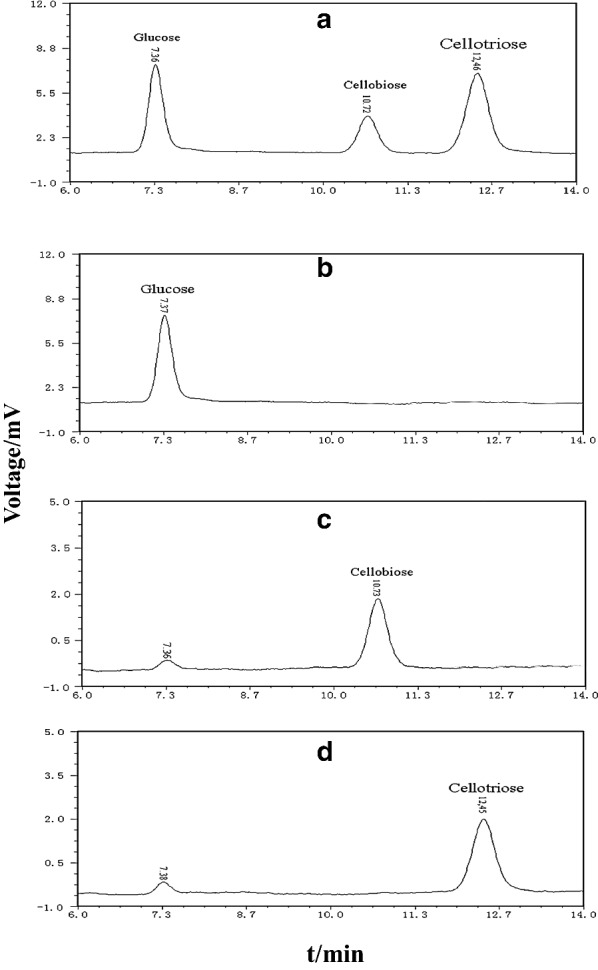
The analysis of transglycosylation activity of the *F. chlamydosporum* HML 278 β-glycosidase. **a** cellobiose + enzyme solution; **b** glucose standard; C: cellobiose standard; **c** cellotriose standard

High performance liquid chromatography analysis showed that BG FH1 hydrolyzed cellobiose (retention time, 10.72 min) to obtain glucose (retention time 7.36 min), which can be further used as a substrate to synthesize cellotriose (retention time 12.46 min) (Fig. 5). The BG FH1 also had transglycoside activity, which was similar to β-glucosidase produced from other strains (Kaya et al. 2008; Seidle et al. 2006; Seidle and Huber 2005).

### The properities of β-glucosidase isolated from ***F. chlamydosporum*** HML278

#### Optimum temperature and thermal stability of β-glucosidase

**Fig. 6:**
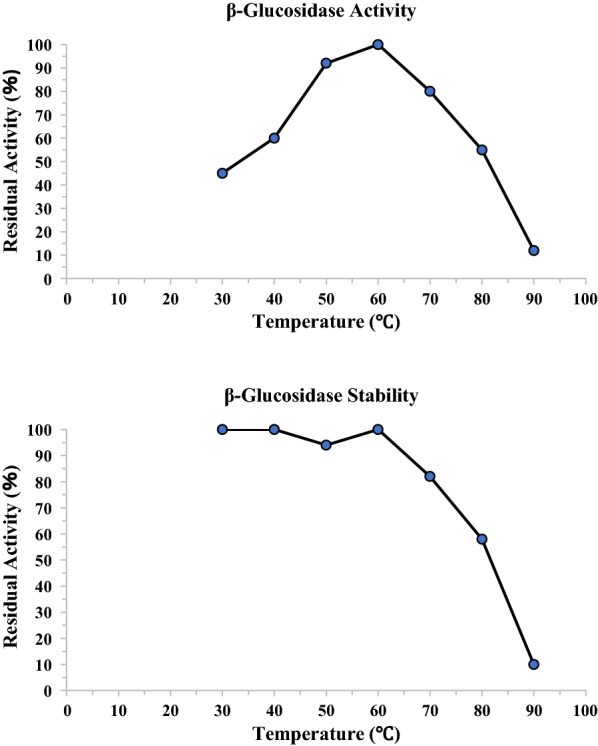
Analysis of the optimum temperature and thermal stability of the β-Glucosidase from *F.chlamydosporum* HML 278

Our results showed that the optimum temperature of β-glucosidases BG FH1 and BG FH2 from *F. chlamydosporium* HML278 was 60 ℃ at pH 5.0. The β-glucosidase exhibited good stability at temperature below 70 ℃, and the enzyme retained 75% of the enzyme activity when incubated at 70 ℃ for 1 hour (Fig. 6). Enzymes that are stable at temperatures above 60 °C are defined as heat-resistant enzymes, and these enzymes play an important role in the production of alcohol by enzymatic saccharification and fermentation of biomass materials (Alani et al. 2008). It has been shown that *Fusarium* species can produce heat-resistant cellulase (Quarantin et al. 2019; Christakopoulos et al. 1995; Kumar et al. 1991; Christakopoulos et al. 1989; Matsumoto et al. 1974; Wood 1969). Our previous study demonstratedthat *F. chlamydosporium* can produce heat-resistant cellulase (Qin et al. 2010).

Chan et al. purified β-glucosidase DT-Bgl and showed that the enzyme exhibited the maxium activity at 70 °C. After hydrolysis of substrate to glucose, which can be further fermented to produce ethanol (Chan et al. 2016). Liew et al. purified a new β-glucosidase BglD5 (GH1) from *Jeotgalibacillus malaysiensis*, and BglD5 was stable at temperature below 65 ℃, which promoted the cellulase hydrolysis (Liew et al. 2018). Kumar found that endoglucanase and β-glucosidase retained enzymatic activity within 60 minutes at 80 °C. These high-temperature enzymes are suitable for application in cellulosic ethanol production (Kumar et al. 1991). Tiwari et al. found that β-glucosidase RA10 from *Bacillus subtilis* was stable at 80 ℃. The heat-stable β-glucosidase enhanced saccharification efficiency and thus released much higher level of glucose than previous reports. This enzyme can enhance the efficiency of hydrolysis and hydrolyze the substance of cellulose into fermentable sugar (Tiwari et al. 2017). Xia et al. found that the cellulase with good thermal stability (stable at 60 ℃) can significantly improve the saccharification efficiency of cellulose hydrolysis (Xia et al. 2016).

Thermophilic fungi can produce heat-stable enzymes. It is of note that cellulose swells at high temperature, which makes it easier to break down. Thus, high temperature can promote the penetration of enzymes into materials and result in a better degradation. The screening of thermophilic fungi and the application of heat-resistant enzymes are important research directions for comprehensive applications of cellulose (Moretti et al. 2012a, b; de Cassia Pereira et al. 2015).

#### The optium pH and stability of purified β-glucosidase at different pH conditions

**Fig. 7:**
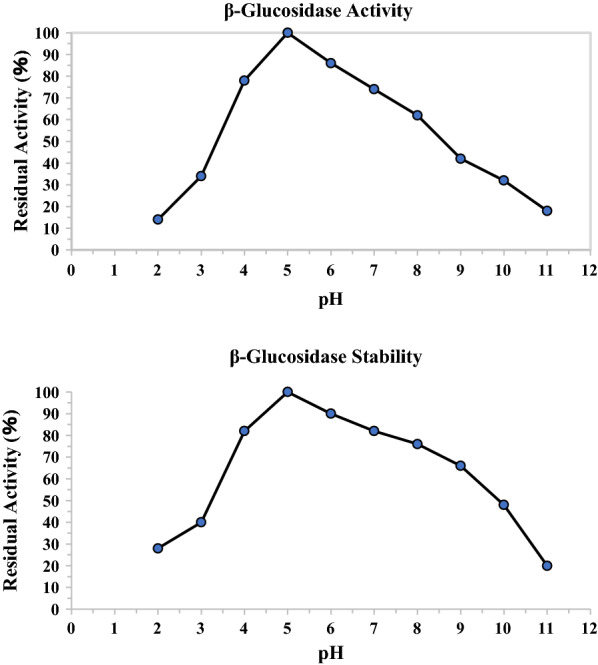
The optimum pH and effect of pH on the activity and stability of the β-Glucosidase from *F.chlamydosporum* HML 278

The β-glucosidases produced by *F. chlamydosporum* HML278 were relatively stable in the pH ranging from 4.0 to 10.0, and showed maximum activity at pH 5.0 (Fig. 7). Christakopoulos et al. screened a strain of *Fusarium oxysporum* and which had an optimum pH of 4.5 (Christakopoulos et al. 1995). Matsumoto et al. screened a *Fusarium moniliforme* strain and the β-glucosidase produced by this strain was stable at pH between 4.0 and 11.0. The enzymes with a wide pH tolerance range usually have broader applications (Matsumoto et al. 1974). Since *F. chlamydosporum* has a wide pH tolerance range, it may have greater application potential (Christakopoulos et al. 1995; Christakopoulos et al. 1989; Matsumoto et al. 1974; Wood 1969).

#### Effect of metal ions on the β-glucosidase purified from *F. chlamydosporum* HML278

Metal ions are often used as activators or inhibitors in the catalytic reaction of enzymes (Grasso et al. 2012). Therefore, adding appropriate metal ions to the enzyme reaction system can improve the catalytic efficiency of the enzyme.

It was shown that Ag^+^, Co^2+^, Cu^2+^, Zn^2+^, and Hg^2+^ strongly inhibited the β-glucosidase purified from *F. chlamydosporum* HML278. In contrast, Mn^2+^, Ca^2+^, Mg^2+^, and Fe^3+^ significantly activated enzyme, whereas Zn^2+^ and Ni^2+^ had no obvious effect on the enzyme activity (Table 2).

**Table 2:** Effect of various metal ions and inhibitors on HML278 β-glucosidase activity

Metal ions and inhibitors	Relative β-glucosidase activity (%)	
10 mM	BG FG1	BG FG2
Control (Crude)	100	100
Mn^2+^	258.6	234.6
Mg^2+^	186.4	176.4
Ca^2+^	159.8	148.2
Zn^2+^	102.4	98.8
Ni^+^	92.6	96.2
Cu^2+^	64.6	54.5
Ag^2+^	56.2	43.4
Co^2+^	48.2	38.2
Hg^2+^	40.8	30.2

Values represent the means of values from three independent experiments, with a standard deviation

It was observed that all divalent metal ions had effects on the enzyme activity. The bivalent ions Hg^2+^ and Co^2+^ completely inhibited enzyme activity. Hg^2+^ can interact with cysteine ​​residues in sulfhydryl bonds (Stricks and Kolthoff  1953). It reacts with cysteine ​​residues, especially in -SH group, and can change the tertiary structure of the protein (Lee et al. 2018). It was speculated that the active site may contain sulfhydryl groups, and these sulfhydryl groups participate in the catalysis and are essential for maintaining the structure of the enzyme (Joo et al. 2009).

The divalent cobalt ion forms a complex with various amino acids, and binding of the cobalt ion to active site of the enzyme is irreversible, completely inhibiting the activity of the enzyme. Other ions, such as Mg^2+^, Mn^2+^, Ca^2+^, Na^+^, Cu^2+^, and Fe^3+^ also tend to form metal complexes with proteins, which ultimately affect enzyme activity by changing protein structure (Shrivastava et al. 2017).

Feng et al. reported that Ca^2+^ increased β-glucosidase Bgl3A activity by 20% (Feng et al. 2015). Xie et al. reported that Ca^2+^ at a concentration of 5 mM increased β-glucosidase activity by 58% (Xie et al. 2015). It has been reported that Ca^2+^ and Mg^2+^ can bind to enzymes to form a stable conformation and improve the catalytic effect (Oyekola et al. 2007).

#### Kinetic experiment of β-glucosidase purified from ***F. chlamydosporum*** HML278

By using pNPG as a substrate, it was shown that the *K*_m_ and *V*_max_ values of β-glucosidase were 2.76 mg/mL and 20.6 U/mg, respectively.

## Discussion

This study reported that *F. chlamydosporum* HML278 can utilize sugarcane bagasse as carbon source for solid-state fermentation to produce heat-resistant β-glucosidase. By employing non-denaturing gel recovery and gel filtration chromatography, two β-glucosidase BG FH1 and BG FH2 were purified, with molecular weights of 93 kDa and 52 kDa, respectively. Purified BG FH1 and BG FH2 were β-glucosidase enzymes with high transglycosidic activity. Thin-layer chromatography and high-performance liquid chromatography analysis showed that BG FH1and BG FH2 had hydrolytic activity and hydrolyzed cellobiose to glucose. Moreover, the enzymes also had transglycosidic activity and can synthesize cellobiose and cellotriose using low molecular weight monosaccharides.

The optimum temperature for purified BG FH1 and BG FH2 from *F. chlamydosporum* HML278 was 60 ℃, and the enzymes were stable below 70 ℃. The enzymes had the highest activity at pH 6.0, and were stable in the pH ranging from pH 4.0 to pH 10.0. Ag^+^, Co^2+^, Cu^2+^, Zn^2+^, and Hg^2+^ had strong inhibitory effect on the purified enzymes, while Mn^2+^, Ca^2+^, Mg^2+^, and Fe^3+^ had obvious activation effect on the enzymes, and Zn^2+^ and Ni^2+^ had no obvious effects on enzymes. In addition, some peptide sequences were also identified by tandem time-of-flight mass spectrometry, but there was no relative information on these peptides in the protein database.

β-glucosidase is a key enzyme that is involved in cellulolytic enzyme-mediated hydrolysis. The presence of sufficient β-glucosidases can also improve the saccharification efficiency of cellulose (Teugjas and Väljamäe 2013; Prawitwong et al. 2013; Ng et al. 2011).

Heat-stable enzymes have obvious advantages as catalysts. Because high temperature can promote the enzyme penetration and cell wall destruction during the process (Kwon et al. 1994), the hydrolysis effect is usually better. Thermophilic fungi are now considered as a promising source for producing thermostable cellulase that used for cellulose degradation, and can increase the saccharification rate (de Cassia Pereira et al. 2015).

By using nuclear magnetic resonance analysis, Makropoulou et al. found that β-glucosidase from *Fusarium oxysporum* had transglycosidic activity. It can catalyze a variety of disaccharides to generate β-D-glucose through transglycosidation. *Fusarium oxysporum* can directly hydrolyze cellulose and synthesize ethanol, glycol, and glycerol after saccharification. Because β-glucosidase has transglycosidic activity, ethanol alcohol is preferentially synthesized (Makropoulou et al. 1998). The cellulase produced by *Fusarium* spp. is heat-resistant. It has been reported that *Fusarium* spp. can also produce enzymes involved in alcohol production, which can saccharify cellulose materials while convert the five- or six-carbon sugars into alcohol (Brunner and Lichtenauer 2007; Gómez-Gómez et al. 2001; Maheshwari et al. 2000; Royer and Moyer 1995; Kumar et al. 1991; Singh and Kumar 1991; Vaidy and Seeta 1984; Wood and McCrae 1977; Sampathnarayanan and Shanmugasundaram 1970). In conclusion, we characterized two β-glucosidases with both hydrolytic and transglycoside activities from *F. chlamydosporum* HML278 fermentation, and these identified enzymes have great potential in industrial application, such as bioethanol, papermaking, feed, food, textile, detergent, and pharmaceutical industries (Xie et al. 2015; Kim et al. 2011; Alani et al. 2008; Maheshwari et al. 2000).

## Availability of data and materials

All data generated or analysed during this study are included in this published article.
